# A Novel Inflammatory and Nutritional Prognostic Scoring System for Nonpathological Complete Response Breast Cancer Patients Undergoing Neoadjuvant Chemotherapy

**DOI:** 10.1155/2022/8044550

**Published:** 2022-12-16

**Authors:** Cong Jiang, Yuting Xiu, Shiyuan Zhang, Xiao Yu, Kun Qiao, Yuanxi Huang

**Affiliations:** Department of Breast Surgery, Harbin Medical University Cancer Hospital, Harbin, China

## Abstract

**Background:**

It has been demonstrated that inflammatory and nutritional variables are associated with poor breast cancer survival. However, some studies do not include these variables due to missing data. To investigate the predictive potential of the INPS, we constructed a novel inflammatory-nutritional prognostic scoring (INPS) system with machine learning.

**Methods:**

This retrospective analysis included 249 patients with malignant breast tumors undergoing neoadjuvant chemotherapy (NAC). After comparing seven potent machine learning models, the best model, Xgboost, was applied to construct an INPS system. K-M survival curves and the log-rank test were employed to determine OS and DFS. Univariate and multivariate analyses were carried out with the Cox regression model. Additionally, we compared the predictive power of INPS, inflammatory, and standard nutritional variables using the *Z* test.

**Results:**

After comparing seven machine learning models, it was determined that the XGBoost model had the best OS and DFS performance (AUC = 0.865 and 0.771, respectively). For overall survival (OS, cutoff value = 0.3917) and disease-free survival (cutoff value = 0.4896), all patients were divided into two groups by the INPS. Those with low INPS had higher 5-year OS and DFS rates (77.2% vs. 50.0%, *P* < 0.0001; and 59.6% vs. 32.1%, *P* < 0.0001, respectively) than patients with high INPS. For OS and DFS, the INPS exhibited the highest AUC compared to the other inflammatory and nutritional variables (AUC = 0.615, *P* = 0.0003; AUC = 0.596, *P* = 0.0003, respectively).

**Conclusion:**

The INPS was an independent predictor of OS and DFS and exhibited better predictive ability than BMI, PNI, and MLR. For patients undergoing NAC for nonpCR breast cancer, INPS was a crucial and comprehensive biomarker. It could also forecast individual survival in breast cancer patients with low HER-2 expression.

## 1. Introduction

Breast malignant tumors, the most common malignancy, now more prevalent than lung cancer worldwide, are the primary cause of cancer-related deaths in women globally [1]. As breast cancer treatment continues to evolve, neoadjuvant chemotherapy (NAC) plays an increasingly important role in determining patient prognosis [2]. To decrease their clinical stage and increase their likelihood of undergoing breast-conserving surgery, NAC is an excellent option for patients with locally advanced breast cancer. Additionally, physicians may now be able to adjust treatments based on drug-sensitivity information [3].

A pathological complete response (pCR) is defined as a breast and lymph node free of invasive cancer on postoperative pathology, but carcinoma in situ of the breast is allowed [4]. Specifically, for the TNBC and HER-2 positive subtypes, achieving a pCR with neoadjuvant therapy predicts an excellent outcome and long-term survival [4, 5]. In contrast, patients with nonpCR breast malignant tumors have a poor prognosis [6]. The CREAT-X trial's findings demonstrated that adjuvant capecitabine therapy could considerably increase OS and DFS in HER-2-negative breast cancer patients who did not achieve a pCR following NAC, with the TNBC group benefiting the most [7]. The KATHERINE study revealed that the 3-year invasive disease-free survival (iDFS) rate of T-DM1 was considerably higher than that of the trastuzumab group for patients who did not achieve a pCR following 6-8 cycles of neoadjuvant therapy [8]. Although the above drugs have improved the prognosis of breast cancer patients with a nonpCR, it is worth considering screening out patients with poor responses to NAC and the standard adjuvant therapy agents and implementing different treatment strategies.

Traditional biomarkers found to be closely connected to a pCR include tumor-infiltrating lymphocytes (TILs), p53, human epidermal growth factor receptor-2 (HER-2), Ki-67 index, estrogen receptor (ER), and progesterone receptor (PR) [9, 10]. However, few biomarkers are specifically designed to predict the outcome of breast cancer patients with a nonpCR. Thus, it is essential and meaningful to construct a novel and convenient biomarker for patients with a nonpCR.

Several studies have recently examined the relationships among inflammation, nutrition, and malignant tumors [11]. Different inflammatory and nutritional parameters, such as body mass index (BMI), prognostic nutrition index (PNI), albumin to globulin ratio (AGR), neutrophil to lymphocyte ratio (NLR), platelet to lymphocyte ratio (PLR), monocyte to lymphocyte ratio (MLR), systemic immune-inflammation index (SII), and systemic inflammation response index (SIRI), as well as their combinations, have all been shown to be vital predictors for breast cancer patients [7–10, 12–15]. However, a single variable can only provide limited information. Compared to models based on one or a few inflammatory indices, prognostic models combining multiple indicators can offer improved prediction accuracy [16, 17].

Biomedicine has embraced machine learning techniques for predictive modeling and decision-making in contrast to conventional statistical methods since they have the potential to produce prediction models by conducting extensive searches across the parameter space [18]. Machine learning methods are more accurate across various subject areas than traditional logistic regression [19].

The above inflammatory and nutritional parameters have attracted extensive attention. However, few studies have comprehensively explored the relationship between these variables and the prognosis, particularly for breast cancer patients receiving neoadjuvant chemotherapy. Therefore, our study is aimed at constructing a novel inflammatory-nutritional scoring (INPS) system based on machine learning models and to investigate its relationship with the outcomes of breast cancer patients with a nonpCR. Then, we compared its predictive ability with commonly used inflammatory and nutritional variables. Additionally, we conducted an exploratory analysis to discuss the relationship between INPS and the HER-2 low expression subtype, as it has become clear from an increasing number of studies that patients with HER-2 low expression breast cancer may have a different prognosis than those with HER-2 negative and positive breast cancer.

## 2. Materials and Methods

### 2.1. Patients

All 249 patients with invasive, malignant breast tumors who underwent NAC and surgery at Harbin Medical University Cancer Hospital between January 2012 and March 2016 were included in the final retrospective analysis. This study was approved by the hospital's ethics committee and complied with the original 1964 Declaration of Helsinki by the World Medical Association and any updated versions. Prior to receiving treatment, each patient signed an informed consent form.

The inclusion criteria included: (1) being diagnosed by pathology with an invasive, malignant breast tumors through core needle biopsy before NAC; (2) undergoing neoadjuvant chemotherapy and surgery at our hospital; (3) available clinical and pathological data, as well as follow-up data; and (4) a postoperative pathology report that indicated that the patient did not achieve a pCR.

The exclusion criteria included (1) achieving a pCR according to the postoperative pathology report; (2) being diagnosed with bilateral breast cancer or other particular types of breast cancer; (3) having distant metastasis; and (4) having an acute or chronic inflammatory disease, such as dermatomyositis.

### 2.2. Classification of Variables

Peripheral venous blood samples were collected seven days before the first cycle of NAC, and the electronic medical records provided all of the patients' clinical and pathological data. The status of nonpCR was evaluated based on the postoperative pathological report.

Patients were divided into groups based on their median age and BMI (according to Chinese standards) [20]. This study used the eighth edition of the TNM staging system from the American Joint Committee on Cancer [21]. Breast cancer is classified into four main subtypes: luminal A, luminal B, HER-2 overexpression (HER2-OE), and triple-negative breast cancer (TNBC) [22]. A HER-2 IHC score of 1+ or 2+ with negative in situ hybridization (ISH) is considered low expression, a HER-2 IHC score of 0 is considered negative, and 3+ or 2+ with positive ISH is considered HER-2 positive [16].

The following parameters were calculated: PNI is serum ALB (g/L) +5 × total lymphocyte count (10^9^/L); AGR is the ratio of albumin to globulin; NLR is the ratio of neutrophil count (10^9^/L) to lymphocyte count (10^9^/L); PLR is the ratio of platelet count (10^9^/L) to lymphocyte count (10^9^/L); MLR is the ratio of monocyte count (10^9^/L) to lymphocyte count (10^9^/L); SII is (neutrophil counts [10^9^/L] × platelet counts [10^9^/L])/total lymphocyte count (10^9^/L). SIRI is (neutrophil counts [10^9^/L] × monocyte counts [10^9^/L))/total lymphocyte count (10^9^/L).

With OS and DFS as the state variables, the maximally selected rank statistics were used to determine the best cutoff values for PNI, AGR, NLR, PLR, MLR, SII, SIRI, lymphocytes (L), neutrophils (N), monocytes (M), hemoglobin (Hb), platelets (P), albumin (ALB), and globulin (GLOB). Then, they were divided into low and high groups according to the following cutoff values: OS.PNI (60.4), OS.AGR (1.24), OS.NLR (2.72), OS.PLR (104), OS.MLR (0.33), OS.SII (672), OS.SIRI (1.4), OS.L (1.48), OS.N (5.9), OS.M (0.36), OS.Hb (132), OS.P (313), OS.ALB (46.3), OS.GLOB (34.8), DFS.PNI (60.4), DFS.AGR (1.24), DFS.NLR (2.47), DFS.PLR (122), DFS.MLR (0.33), DFS.SII (672), DFS.SIRI (1.19), DFS.L (1.39), DFS.N (5.47), DFS.M (0.36), DFS.Hb (132), DFS.P (304), DFS.ALB (43), and DFS.GLOB (28.5).

### 2.3. Follow-Up

Patients were followed up every three months after surgery for the first two years and then every six months for the following three years. Follow-up was up to five years after surgery or the date of death from any cause. OS was defined as the time between the date of operation and the date of death from any cause or last follow-up, and DFS was defined as the time from the date of surgery to the date of metastasis to distant organs, local recurrence, or death from any cause.

### 2.4. Machine Learning, Inflammatory and Nutritional Variables

Seven robust machine learning models were used to predict OS and DFS, including logistic regression (LR), support vector classification (SVC), k-nearest neighbor classification (KNN), extreme gradient boosting (Xgboost), random forests (RF), light gradient boosting machine (LightGBM), and adaptive boosting (AdaBoost). This study adopted the hold-out method (simple cross-validation) to address the overfitting issue brought on by the small sample size. The performance of each model was compared through the area under the curve (AUC) of the receiver operating characteristic (ROC). The most effective machine learning model was used to determine the importance of the inflammatory and nutritional variables as features.

### 2.5. Statistical Analysis

Statistical analyses were conducted with Python (version 3.9), R software (version 3.6.1), and MedCalc software (version 19.0.7). The cutoff values of the INPS and hematological variables were determined by the maximally selected rank statistics through the maxstat.text function based on the “maxstat” package in R software [17], with an initial cutoff score of 1 being assigned to variables above the cutoff value and an initial score of 0 to variables below it. Frequencies and percentages (%) were applied to describe the categorical variables, while the chi-squared test or Fisher's exact test were used to assess differences. The median value of the continuous variables is presented with the interquartile range (IQR). The multicollinearity relationship among INPS, inflammatory and nutritional variables was tested by multiple linear regression analysis via variance inflation factor (VIF), with a VIF ≤ 2 considered noncollinear [23]. The Kaplan–Meier method was employed to estimate the survival curves, which were then compared by the log-rank test. The independent prognostic factors were determined with the Cox proportional hazards model, and pH assumptions were checked by the log minus log (LML) survival function. The *Z* test was used to compare different groups' predictive functions, with a *P* value <0.05 indicating statistical significance.

## 3. Results

### 3.1. Construction of INPS

**Figure 1:**
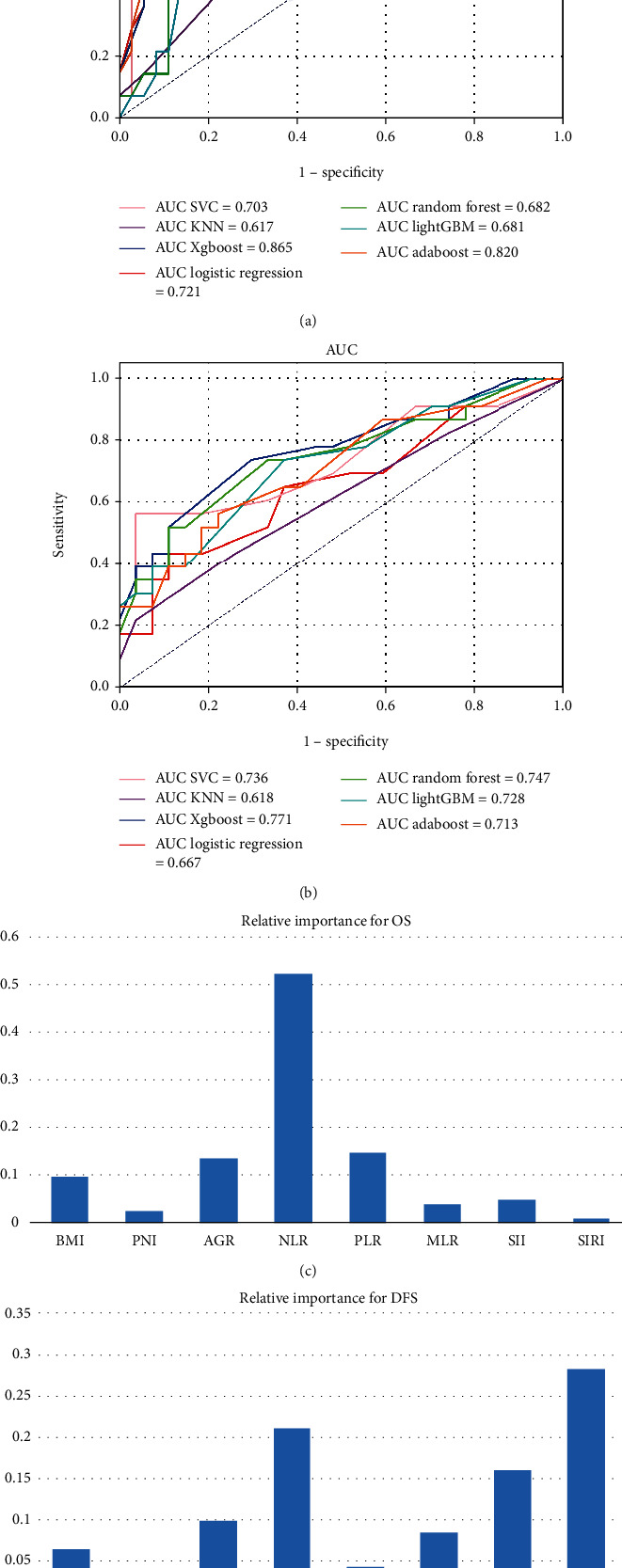
The comparison of different machine learning models performance (a, b), and the feature importance of different inflammatory and nutritional variables in predicting OS and DFS based on Xgboost model (c, d).

**Figure 2:**
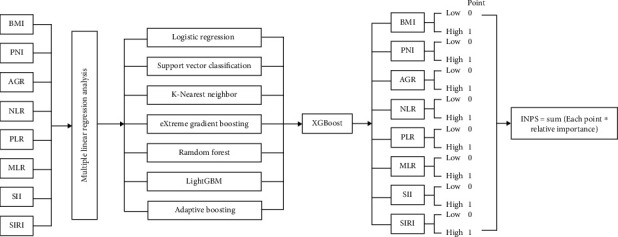
The flow chart of INPS construction.

Multiple linear regression analysis was conducted to test the possibility of multicollinearity between the inflammatory and nutritional variables, which showed that all of the variables had a VIF ≤ 2. Eight inflammatory and nutritional variables were included in the seven machine-learning models to predict OS and DFS. The Xgboost model exhibited the highest AUC compared to other models for predicting OS or DFS (AUC = 0.865 and 0.771, respectively, Figures 1(a) and 1(b)). Then, the relative importance of the inflammatory and nutritional variables for predicting OS and DFS was calculated using the Xgboost model (Figures 1(c) and 1(d)). Variables below the respective cutoff value were scored 0, and those above the cutoff value were scored 1. The INPS was calculated as follows: OS.INPS = sum (the score of each inflammatory and nutritional variable × respective relative importance for OS), DFS. INPS = sum (the score of each inflammatory and nutritional variable × respective relative importance for DFS) (Figure 2). According to the maxstat.text function, all of the patients were divided into low and high groups with a cutoff value for OS.INPS (0.3917) and DFS.INPS (0.4896).

### 3.2. Differences in Clinical and Pathologic Variables for Different INPS Groups

All 249 nonpCR breast malignant tumor patients were divided into two groups by the cutoff values of OS.INPS (0.3917) and DFS.INPS (0.4896). There were 193 (77.5%) cases in the low INPS group and 56 (22.5%) cases in the high INPS group, despite the state variable of OS or DFS, with ages ranging from 22 to 72 years old (median: 49 years old). A total of 217 (87.1%) patients suffered from clinical TNM stage III, and 117 (47.0%) patients suffered from the luminal B subtype. Clinical T stage, Ki-67 index, L, N, M, PNI, NLR, PLR, MLR, SII, and SIRI were correlated with OS.INPS status (*P* < 0.05), while clinical T stage, HER-2 status, Ki-67 index, P53, L, N, M, PNI, NLR, PLR, MLR, SII, and SIRI were correlated with DFS.INPS status (*P* < 0.05) (Tables 1 and 2).

### 3.3. Univariable and Multivariable cox Regression Analysis for OS and DFS

**Figure 3:**
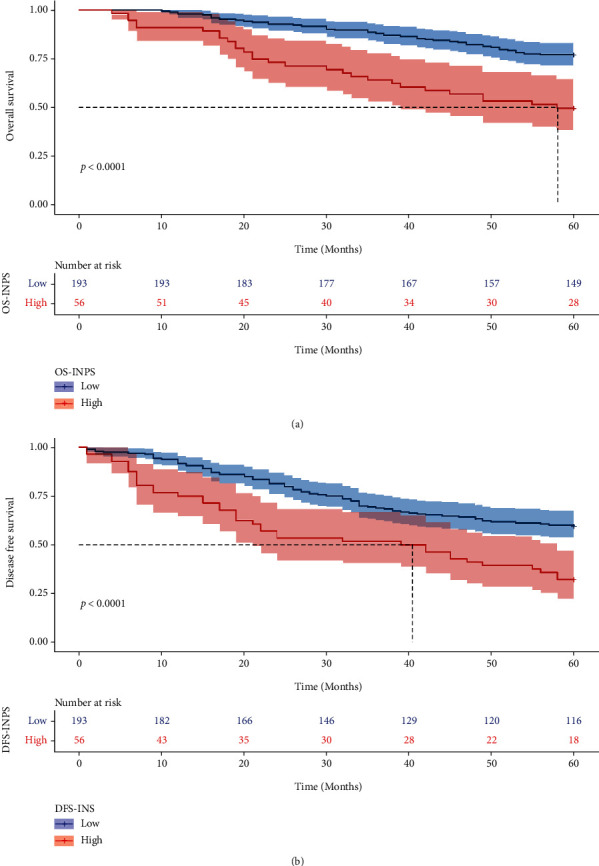
Kaplan-Meier curves of different INPS groups for OS (a) and DFS (b).

The multicollinearity between INPS, inflammatory and nutritional variables was tested prior to the Cox analysis. OS.INPS, OS.NLR, OS.SII, and OS.SIRI had a VIF value of >2 for the state variable of OS. DFS.INPS, DFS.NLR, DFS.SII, and DFS.SIRI had a VIF of >2 for the state variable of DFS. Additionally, the INPS was constructed based on these inflammatory and nutritional variables. Therefore, the Cox regression analysis excluded BMI, PNI, AGR, NLR, PLR, MLR, SII, and SIRI. The relationship between the inflammatory and nutritional variables and OS and DFS is illustrated in Table S1. Meanwhile, the pH assumptions were checked using the log minus log (LML) survival function, and the Cox regression model was appropriate for the study data. In univariable Cox analysis, parturition, OS.INPS, OS.N, and clinical T stage were predictors of OS, while parturition, DFS.INPS, DFS.N, and DFS.M were predictors of DFS. Variables with *P* < 0.05 were included in the multivariate analysis, demonstrating that parturition, OS.INPS and clinical T stage were independently associated with OS (*P* = 0.006, HR: 0.41, 95% CI: 0.22-0.77; *P* < 0.001, HR: 2.41, 95% CI: 1.45-4.01; *P* = 0.014, HR: 5.70, 95% CI: 1.43-22.8, respectively, Table 3). Only parturition and DFS.INPS were independently associated with DFS (*P* = 0.003, HR: 0.45, 95% CI: 0.27-0.76; *P* = 0.005, HR: 1.84, 95% CI: 1.20-2.83, respectively, Table 4). Compared to the high OS.INPS and high DFS.INPS groups, the low OS.INPS and low DFS.INPS groups exhibited higher 5-year OS and DFS rates (77.2% vs. 50.0%, *P* < 0.0001; 59.6% vs. 32.1%, *P* < 0.0001, respectively, Figures 3(a) and 3(b)). In addition, the mean OS and DFS in the low INPS groups was significantly prolonged compared with that in the high INPS groups (54 vs. 43 months, *P* < 0.0001; 46 months vs. 35 months, *P* < 0.0001, respectively, Figures 3(a) and 3(b)).

### 3.4. Relationships among OS, DFS, and INPS in Breast Cancer Patients with Different Clinical T Stages

Tables 1 and 2 reveal that clinical T stage, HER-2 status, Ki-67, and P53 were significantly related to INPS. Therefore, we conducted an exploratory analysis in these subgroups to identify the predictive ability of INPS for OS and DFS.

**Figure 4:**
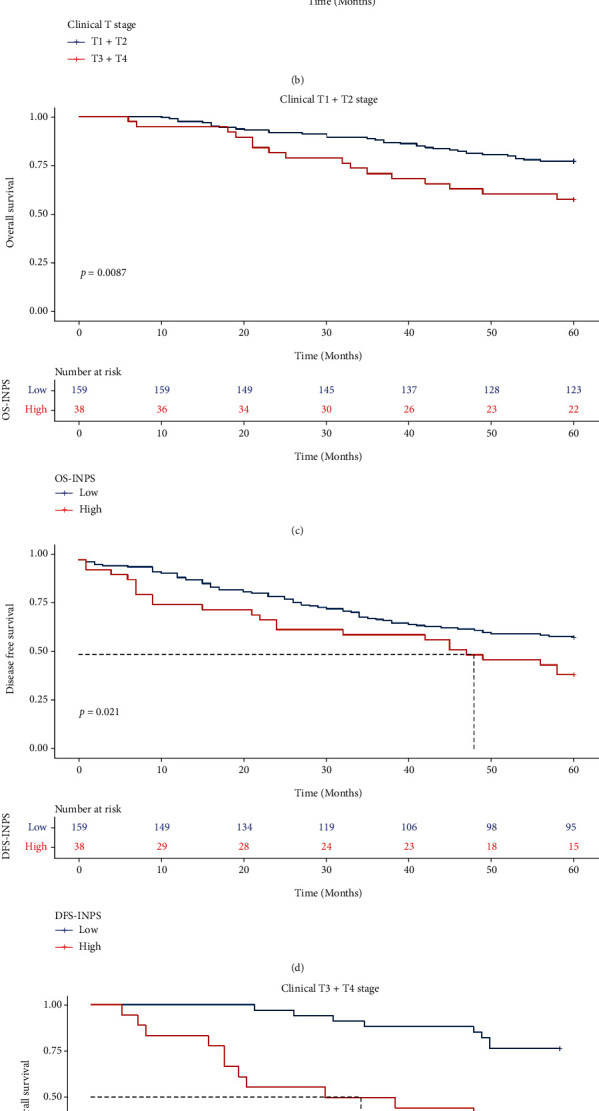
Kaplan–Meier curves of different clinical T stage groups for OS and DFS. K-M analysis of OS (a) and DFS (b) for breast cancer patients by clinical T stage; K-M analysis of OS (c) and DFS (d) for breast cancer patients of T1 + T2 by INPS; K-M analysis of OS (e) and DFS (f) for breast cancer patietns of T3 + T4 by INPS.

In all of the nonpCR breast cancer patients, compared to the clinical T3 + T4 group, patients with clinical T1 + T2 stage disease showed higher 5-year OS and DFS rates (73.6% vs. 61.5%, *X*^2^ = 3.192, *P* = 0.074; 55.3% vs. 46.2%, *X*^2^ = 1.604, *P* = 0.21, respectively, Figures 4(a) and 4(b)). In the clinical T1 + T2 subgroup, patients with low INPS had significantly higher 5-year OS and DFS rates than those with high INPS (77.4% vs. 57.9%, *X*^2^ = 6.9, *P* = 0.0087; 59.1% vs. 39.5%, *X*^2^ = 5.3, *P* = 0.021, respectively, Figures 4(c) and 4(d)). In the clinical T3 + T4 subgroup, patients with low INPS also had significantly improved 5-year OS and DFS rates (76.5% vs. 33.3%, *X*^2^ = 13.3, *P* = 0.00026; 61.8% vs. 16.7%, *X*^2^ = 15.6, *P* < 0.0001, respectively, Figures 4(e) and 4(f)).

### 3.5. Relationships among OS, DFS, and INPS in Breast Cancer Patients with Different HER-2 Statuses

**Figure 5:**
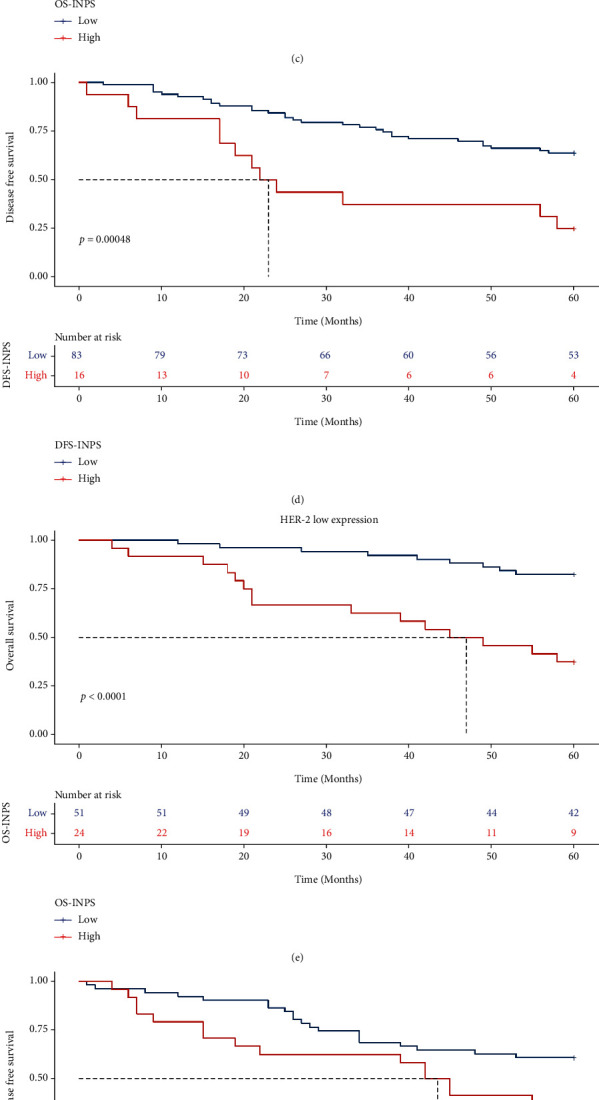
Kaplan-Meier curves of different HER-2 status breast cancer patients for OS (a) DFS (b). K-M analysis of OS (c) and DFS (d) for HER-2 negative breast cancer patients by INPS; K-M analysis of OS (e) and DFS (f) for HER-2 low expression breast cancer patients by INPS; K-M analysis of OS (g) and DFS (h) for HER-2 positive breast cancer patients by INPS.

In all breast cancer patients, there was no distinct difference in the 5-year OS and DFS rates among the HER-2-negative, low expression, and positive subgroups (69.7% vs. 68.0% vs. 76.0%, *X*^2^ = 1.1, *P* = 0.58; 57.6% vs. 52.0% vs. 49.3%, *X*^2^ = 1.7, *P* = 0.42; Figures 5(a) and 5(b)). In the HER-2-negative subgroup, patients in the low INPS group had significantly higher 5-year OS and DFS rates than those in the high INPS group (75.0% vs. 47.4%, *X*^2^ = 7.8, *P* = 0.0051; 63.9% vs. 25.0%, *X*^2^ = 12.2, *P* = 0.00048, respectively, Figures 5(c) and 5(d)). In the HER-2 low expression subgroup, patients in the low INPS groups also had significantly higher 5-year OS and DFS rates (82.4% vs. 37.5%, *X*^2^ = 18.0, *P* < 0.0001; 60.8% vs. 33.3%, *X*^2^ = 4.9, *P* = 0.026, respectively, Figures 5(e) and 5(f)). In the HER-2-positive subgroup, there was no distinct difference in OS and DFS between the low and high INPS groups (75.8% vs. 76.9%, *X*^2^ = 0.000, *P* = 0.99; 52.5% vs. 37.5%, *X*^2^ = 2.0, *P* = 0.16, respectively, Figures 5(g) and 5(h)).

### 3.6. Relationships among OS, DFS, and INPS in Breast Cancer Patients with Different Ki-67 Indices

**Figure 6:**
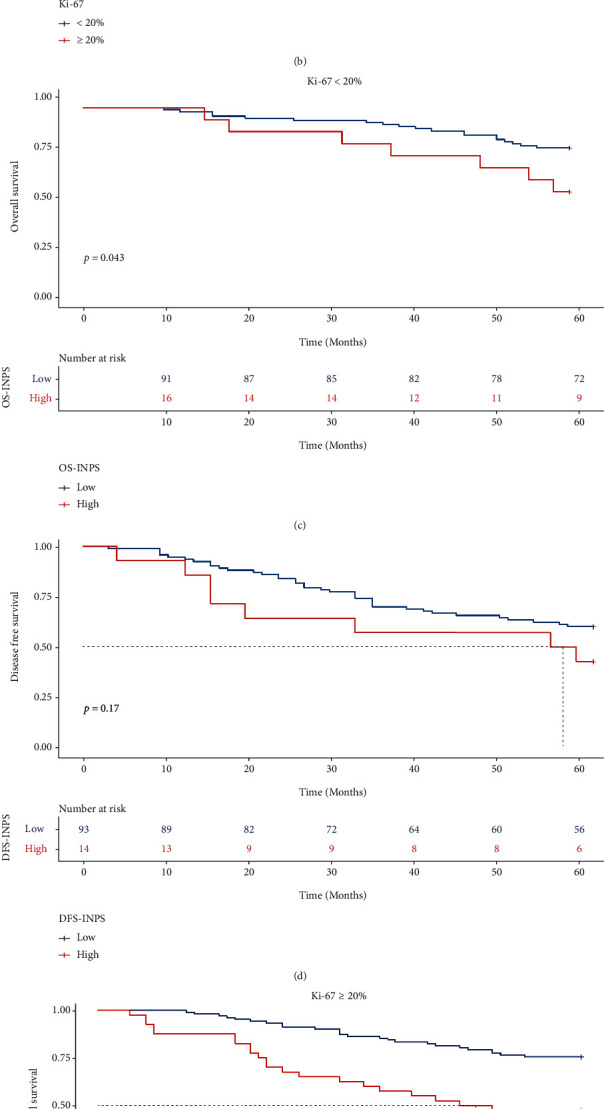
Kaplan-Meier curves of different Ki-67 index breast cancer patients for OS (a) DFS (b). K-M analysis of OS (c) and DFS (d) for breast cancer patients with Ki-67<20% by INPS; K-M analysis of OS (e) and DFS (f) for breast cancer patients with Ki-67 ⩾ 20% by INPS.

In all breast cancer patients, no distinct difference was observed in the 5-year OS and DFS rates between the Ki-67<20% and Ki-67 ≥ 20% groups (75.7% vs. 67.6%, *X*^2^ = 2.6, *P* = 0.11; 57.9% vs. 50.0%, *X*^2^ = 2.1, *P* = 0.15, respectively, Figures 6(a) and 6(b)). However, in the Ki-67<20% subgroup, patients with low OS.INPS had a higher 5-year OS rate than the high OS.INPS group (79.1% vs. 47.2%, *X*^2^ = 4.1, *P* = 0.043; Figure 6(c)), with no difference in the 5-year DFS rate between the low and high DFS.INPS groups (60.2% vs. 42.9%, *X*^2^ = 1.9, *P* = 0.17; Figure 6(d)). In the Ki-67 ≥ 20% group, patients with low INPS had significantly higher 5-year OS and DFS than those with high INPS (75.5% vs. 47.5%, *X*^2^ = 13.4, *P* = 0.00026; 59.0% vs. 28.6%, *X*^2^ = 12.9, *P* = 0.00033, respectively, Figures 6(e) and 6(f)).

### 3.7. Relationships among OS, DFS, and INPS in Breast Cancer Patients with Different P53 Statuses

**Figure 7:**
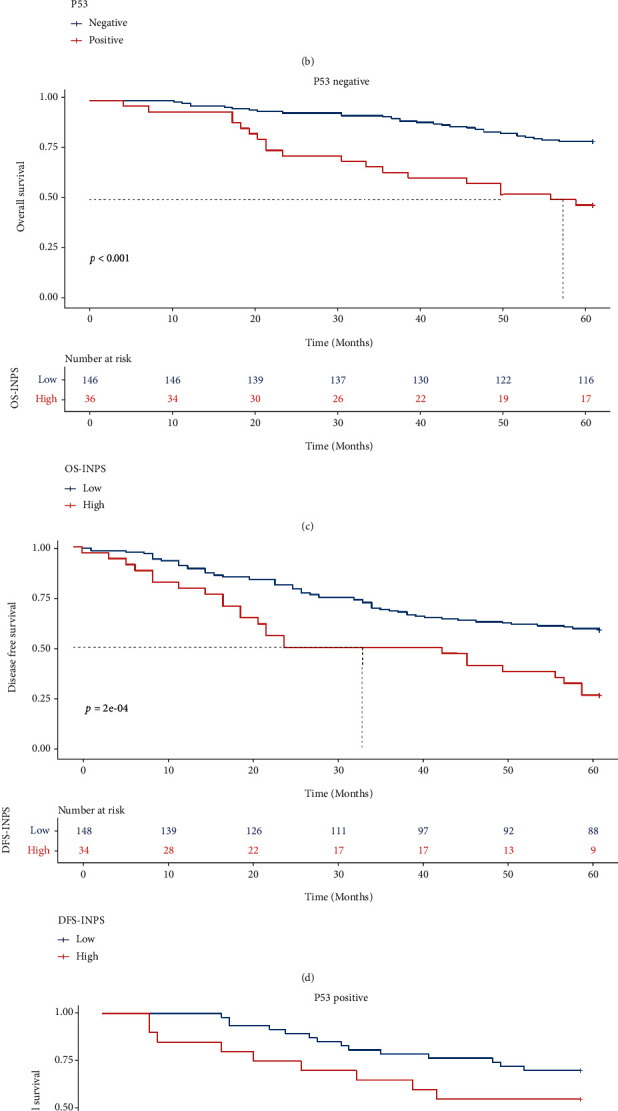
Kaplan-Meier curves of different P53 status breast cancer patients for OS (a) DFS (b). K-M analysis of OS (c) and DFS (d) for P53 negative breast cancer patients by INPS; K-M analysis of OS (e) and DFS (f) for P53 positive breast cancer patients by INPS.

In all malignant breast cancer patients, there was no difference in 5-year OS and DFS rates between the P53-negative and P53-positive groups (73.1% vs. 65.7%, *X*^2^ = 1.8, *P* = 0.018; 52.7% vs. 55.2%, *X*^2^ = 0.031, *P* = 0.86, respectively, Figures 7(a) and 7(b)). In the P53-negative group, patients with a low INPS showed significantly higher 5-year OS and DFS rates (79.5% vs. 47.2%, *X*^2^ = 18.9, *P* < 0.0001; 58.8% vs. 26.5%, *X*^2^ = 13.8, *P* < 0.0001, respectively, Figures 7(c) and 7(d)). However, in the P53-positive group, there was no difference in the 5-year OS rate between the low and high OS.INPS groups (70.2% vs. 55.0%, *X*^2^ = 2.1, *P* = 0.15; Figure 7(e)), while patients with low DFS.INPS had a higher 5-year DFS rate than those in the high DFS.INPS group (62.2% vs. 40.9%, *X*^2^ = 4.0, *P* = 0.045; Figure 7(f)).

### 3.8. Comparison of the Predictive Capacity of INPS, Inflammatory and Nutritional Variables

**Figure 8:**
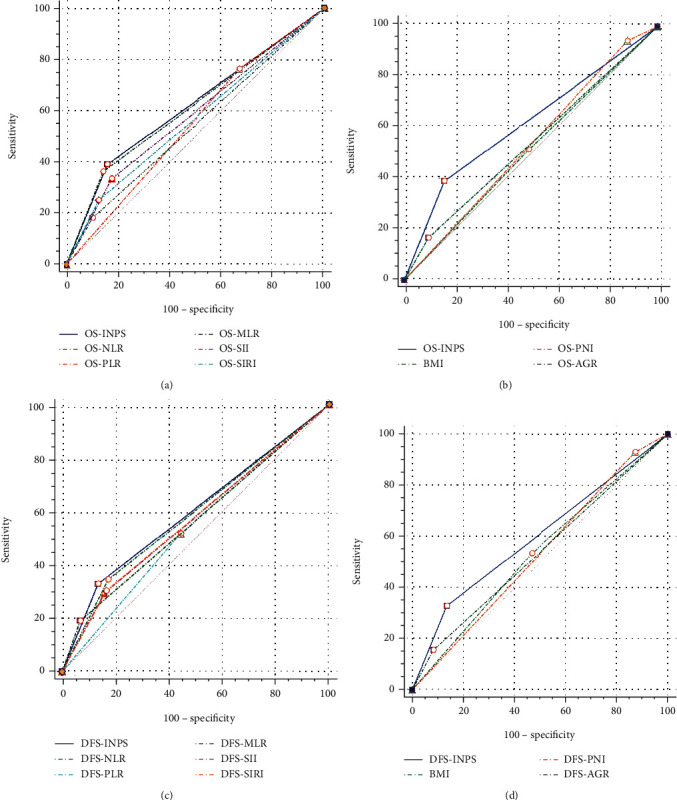
The comparison of prognostic ability between INPS, inflammatory and nutritional variables for OS (a, b) and DFS (c, d).

The AUC was compared using the *Z* test to evaluate the prognostic significance of the INPS and inflammatory and nutritional variables. Whether the state variable was OS or DFS, INPS had the highest AUC compared with the other inflammatory and nutritional variables (AUC = 0.615, *P* = 0.0003; AUC = 0.596, *P* = 0.0003, respectively, Table 5, Figure 8). Meanwhile, the distinction of AUC between OS.INPS and OS.BMI (*Z* = 2.094, 95% CI: 0.007-0.202, *P* = 0.0363), OS.INPS and OS.PNI (*Z* = 2.467, 95% CI: 0.017-0.150, *P* = 0.0136), OS.INPS and OS.MLR (*Z* = 2.603, 95% CI: 0.019-0.133, *P* = 0.0092), OS.NLR and OS.MLR (*Z* = 2.516, 95% CI: 0.016-0.125, *P* = 0.0119), and DFS.INPS and DFS.PNI (*Z* = 2.193, 95% CI: 0.007-0.126, *P* = 0.0283) were statistically significant (Table 6). There were no distinct differences between any other groups (*P* > 0.05).

## 4. Discussion

This study investigated the clinical significance of a novel inflammatory-nutritional prognostic scoring (INPS) system based on BMI, PNI, AGR, NLR, PLR, MLR, SII, and SIRI through machine learning for breast cancer patients with a nonpCR after undergoing NAC and surgery. Low INPS was significantly associated with prolonged OS and DFS. This study also compared the predictive ability of INPS with the common inflammatory and nutritional variables, revealing that INPS was a better predictor for OS and DFS. The exploratory analysis demonstrated that INPS was a promising biomarker for HER-2 negative and low expression breast cancer patients.

Studies have shown that malignant tumors are related to systemic inflammation [24, 25]. Cancer-related inflammation occurs when cancer and inflammatory responses are entangled, resulting in a dramatically poor prognosis and a failure to respond to cancer therapy [11]. As a part of the inflammatory parameters, neutrophils may promote proliferation and metastasis by releasing inflammatory mediators [26]. Monocytes are also correlated with the metastasis and progression of malignant tumors [27]. In contrast, lymphocytes are essential for the antitumor effect [28]. Additionally, malnutrition is associated with cancer progression, as it may cause a poor immune response [29]. As a manifestation of malnutrition, poor survival is associated with low serum albumin levels [30].

As a holistic variable that incorporates many common inflammatory and nutritional variables, the utility of the INPS has been explored in other malignant tumors. Wang et al. found that preoperative INPS is an independent predictor of outcomes for stage III GC patients [31]. Hua et al. demonstrated that patients with high INPS had significantly worse survival than those with low INPS [32]. In that research, the authors chose the LASSO regression model to establish the INPS. However, as the LASSO algorithm is a type of machine learning, selection bias could not be avoided despite a large study sample. Therefore, in our study, we compared seven standard machine learning algorithms and selected the best model, Xgboost (AUC = 0.865 and 0.771, respectively, Figures 1(a) and 1(b)), to construct the INPS for OS and DFS. The multivariable Cox analysis demonstrated that OS. INPS and DFS. INPS were all independent predictors of outcomes for nonpCR breast cancer patients undergoing NAC and surgery (*P* < 0.001, HR: 2.41, 95% CI: 1.45-4.01; *P* = 0.005, HR: 1.84, 95% CI: 1.20-2.83; Tables 3 and 4, respectively).

Many studies have demonstrated that inflammatory and nutritional parameters are associated with survival; however, some of their results are inconsistent. According to a meta-analysis, a high NLR was significantly correlated with a poor pathological response in breast malignant tumor patients, with no association found with DFS or OS [33]. In contrast, another meta-analysis found that patients with high NLR and PLR had short OS and an increased risk of recurrence [34]. In addition, compared with NLR, which could only offer limited clinical information, our results noted that SII, an inflammatory parameter composed of neutrophils, platelets, and lymphocytes, was a better predictor of OS [9]. Therefore, we assumed that a biomarker integrated with various inflammatory and nutritional parameters should be more accurate than an individual biomarker. Our results proved that the INPS had a higher AUC for OS and DFS than the other inflammatory and nutritional variables. Pairwise comparisons of INPS, inflammatory and nutritional variables and the results of the *Z* test revealed that OS.INPS had a significantly larger AUC than OS.BMI, OS.PNI, and OS.MLR, and DFS.INPS had a substantially larger AUC than DFS.PNI.

We also conducted an exploratory analysis in patients with different clinical T stages, HER-2 statuses, Ki-67 indices, and P53 levels. Although significant survival differences could not be found in among above subgroups, patients with different INPSs showed considerable differences in OS and DFS. Especially in the distinct HER-2 status subgroups, patients with low INPS had better OS and DFS in HER-2 negative and low expression subgroups, with no difference observed in the HER-2 positive group. More recent studies have shown that breast cancer patients with low HER-2 expression have improved 3-year OS and DFS compared to HER-2-negative patients [35]. However, it is unclear whether low HER-2 expression is correlated with the long-term prognosis in breast cancer patients. Thus, the INPS may be a promising biomarker for HER-2 low breast cancer patients.

Although comprehensive and novel, this study had some limitations. First, it was a retrospective analysis conducted in a single center, and validation with data from additional centers may be necessary. Second, a more extended follow-up period is necessary to identify the long-term clinical significance of INPS. Last, the dynamic changes in INPS should be explored to identify its predictive ability more fully.

## 5. Conclusions

For nonpCR breast cancer patients receiving NAC, the INPS based on eight common inflammatory and nutritional variables is an independent predictor of survival. As a comprehensive parameter, it is superior to BMI, PNI, and MLR in predicting survival time. Additionally, it may be a promising biomarker for breast cancer with low HER-2 expression.

## Figures and Tables

**Table 1:** Clinical and pathological characteristics divided by OS-INPS.

Variables	*n* = 249	OS − INPS ≤ 0.3917	OS − INPS > 0.3917	*P*
		*n* = 193 (%)	*n* = 56 (%)	
Age (median (IQR))	49.00(42.00-57.00)	49.00(42.00-57.00)	47.00 (42.75-56.25)	0.507
Age				0.392
≤ 49	132 (53.0)	99 (51.3)	33 (58.9)	
> 49	117 (47.0)	94 (48.7)	23 (41.1)	
Position				1
Left	140 (56.2)	109 (56.5)	31 (55.4)	
Right	109 (43.8)	84 (43.5)	25 (44.6)	
Menopause				0.083
No	137 (55.0)	100 (51.8)	37 (66.1)	
Yes	112 (45.0)	93 (48.2)	19 (33.9)	
Parturition				0.164
0	31 (12.4)	20 (10.4)	11 (19.6)	
1	156 (62.7)	125 (64.8)	31 (55.4)	
≥ 2	62 (24.9)	48 (24.9)	14 (25.0)	
Clinical T stage				**0.019**
1	22 (8.8)	16 (8.3)	6 (10.7)	
2	175 (70.3)	143 (74.1)	32 (57.1)	
3	48 (19.3)	33 (17.1)	15 (26.8)	
4	4 (1.6)	1 (0.5)	3 (5.4)	
Clinical N stage				0.815
0	8 (3.2)	6 (3.1)	2 (3.6)	
1	28 (11.2)	22 (11.4)	6 (10.7)	
2	153 (61.4)	116 (60.1)	37 (66.1)	
3	60 (24.1)	49 (25.4)	11 (19.6)	
Clinical TNM stage				0.752
II	32 (12.9)	26 (13.5)	6 (10.7)	
III	217 (87.1)	167 (86.5)	50 (89.3)	
Molecular subtype				0.302
Luminal A	39 (15.7)	33 (17.1)	6 (10.7)	
Luminal B	117 (47.0)	86 (44.6)	31 (55.4)	
HER-2 OE	50 (20.1)	42 (21.8)	8 (14.3)	
TNBC	43 (17.3)	32 (16.6)	11 (19.6)	
ER				0.632
Negative	98 (39.4)	78 (40.4)	20 (35.7)	
Positive	151 (60.6)	115 (59.6)	36 (64.3)	
PR				0.206
Negative	123 (49.4)	100 (51.8)	23 (41.1)	
Positive	126 (50.6)	93 (48.2)	33 (58.9)	
HER-2				0.059
Negative	99 (39.8)	80 (41.5)	19 (33.9)	
Low expression	75 (30.1)	51 (26.4)	24 (42.9)	
Positive	75 (30.1)	62 (32.1)	13 (23.2)	
Ki-67				**0.020**
<20%	107 (43.0)	91 (47.2)	16 (28.6)	
≥ 20%	142 (57.0)	102 (52.8)	40 (71.4)	
P53				0.129
Negative	182 (73.1)	146 (75.6)	36 (64.3)	
Positive	67 (26.9)	47 (24.4)	20 (35.7)	
Cycle				0.808
≤ 4	148 (59.4)	116 (60.1)	32 (57.1)	
>4	101 (40.6)	77 (39.9)	24 (42.9)	
L (median (IQR))	1.92 (1.60-2.45)	2.15 (1.75-2.62)	1.56 (1.37-1.80)	**<0.001**
N (median (IQR))	3.78 (3.00-4.73)	3.42 (2.86-4.21)	5.06 (4.51-6.07)	**<0.001**
M (median (IQR))	0.41 (0.34-0.52)	0.39 (0.31-0.50)	0.47 (0.40-0.57)	**<0.001**
Hb (median (IQR))	135.0(128.30-141.00)	135.50(129.6-141.0)	130.8 (125.9-140.3)	0.111
P (median (IQR))	239(210-283)	236(209-278)	260 (212.0-300.5)	0.115
ALB (median (IQR))	45.00 (43.00-46.50)	45.00 (43.00-46.30)	45.00 (43.00-46.62)	0.386
GLOB (median (IQR))	30.00 (27.60-33.00)	30.00 (27.30-33.00)	30.00 (27.98-33.62)	0.494
BMI (median (IQR))	24.00 (21.80-26.70)	24.00 (21.70-26.60)	23.90 (21.98-27.10)	0.522
PNI (median (IQR))	55.10 (52.30-57.50)	55.50 (52.60-58.10)	53.05 (50.60-55.30)	**<0.001**
AGR (median (IQR))	1.48 (1.35-1.64)	1.49 (1.38-1.63)	1.47 (1.33-1.67)	0.699
NLR (median (IQR))	1.87 (1.45-2.47)	1.66 (1.37-2.03)	3.20 (2.86-3.64)	**<0.001**
PLR (median (IQR))	120(98-150)	112(93-135)	165(134-206)	**<0.001**
MLR (median (IQR))	0.20 (0.16-0.27)	0.19 (0.15-0.22)	0.30 (0.25-0.36)	**<0.001**
SII (median (IQR))	441 (329-630)	397.00(299-508)	813(696-1001)	**<0.001**
SIRI (median (IQR))	0.77 (0.51-1.14)	0.64 (0.48-0.87)	1.50 (1.20-2.08)	**<0.001**

Abbreviations: INPS, inflammation and nutrition prognostic score; IQR, interquartile range; ER, estrogen receptor; PR, progesterone receptor; HER-2, human epidermal growth factor receptor 2; HER-2 OE, HER-2 overexpression; L, lymphocyte; N, neutrophil; M, monocyte; Hb, hemoglobin; P, platelet; ALB, albumin; GLOB, globulin; BMI, body mass index; PNI, prognostic nutritional index; AGR, albumin-globulin ratio; NLR, neutrophil-lymphocyte ratio; PLR, platelet-lymphocyte ratio; MLR, monocyte-lymphocyte ratio; SII, systemic immune inflammation index; SIRI, system inflammation response index.

**Table 2:** Clinical and pathological characteristics divided by DFS-INPS.

Variables	*n* = 249 (%)	DFS − INPS ≤ 0.4896	DFS − INPS > 0.4896	*P*
		*n* = 193 (%)	*n* = 56 (%)	
Age (median (IQR))	49.00 (42.00-57.00)	49.00 (42.00-57.00)	47.50 (42.00-57.00)	0.904
Age				
≤ 49	132 (53.0)	102 (52.8)	30 (53.6)	1
> 49	117 (47.0)	91 (47.2)	26 (46.4)	
Position				
Left	140 (56.2)	110 (57.0)	30 (53.6)	0.763
Right	109 (43.8)	83 (43.0)	26 (46.4)	
Menopause				
0	137 (55.0)	102 (52.8)	35 (62.5)	0.26
1	112 (45.0)	91 (47.2)	21 (37.5)	
Parturition				
0	31 (12.4)	21 (10.9)	10 (17.9)	0.219
1	156 (62.7)	126 (65.3)	30 (53.6)	
≥ 2	62 (24.9)	46 (23.8)	16 (28.6)	
Clinical T stage				
1	22 (8.8)	17 (8.8)	5 (8.9)	**0.026**
2	175 (70.3)	142 (73.6)	33 (58.9)	
3	48 (19.3)	33 (17.1)	15 (26.8)	
4	4 (1.6)	1 (0.5)	3 (5.4)	
Clinical N stage				
0	8 (3.2)	6 (3.1)	2 (3.6)	0.715
1	28 (11.2)	23 (11.9)	5 (8.9)	
2	153 (61.4)	115 (59.6)	38 (67.9)	
3	60 (24.1)	49 (25.4)	11 (19.6)	
Clinical TNM stage				
II	32 (12.9)	27 (14.0)	5 (8.9)	0.442
III	217 (87.1)	166 (86.0)	51 (91.1)	
Molecular subtype				
Luminal A	39 (15.7)	34 (17.6)	5 (8.9)	0.165
Luminal B	117 (47.0)	86 (44.6)	31 (55.4)	
HER-2 OE	50 (20.1)	42 (21.8)	8 (14.3)	
TNBC	43 (17.3)	31 (16.1)	12 (21.4)	
ER				
Negative	98 (39.4)	77 (39.9)	21 (37.5)	0.867
Positive	151 (60.6)	116 (60.1)	35 (62.5)	
PR				
Negative	123 (49.4)	98 (50.8)	25 (44.6)	0.511
Positive	126 (50.6)	95 (49.2)	31 (55.4)	
HER-2				
Negative	99 (39.8)	83 (43.0)	16 (28.6)	**0.045**
Low expression	75 (30.1)	51 (26.4)	24 (42.9)	
Positive	75 (30.1)	59 (30.6)	16 (28.6)	
Ki-67				
<20%	107 (43.0)	93 (48.2)	14 (25.0)	**0.003**
≥ 20%	142 (57.0)	100 (51.8)	42 (75.0)	
P53				
Negative	182 (73.1)	148 (76.7)	34 (60.7)	**0.028**
Positive	67 (26.9)	45 (23.3)	22 (39.3)	
Cycle				
≤ 4	148 (59.4)	117 (60.6)	31 (55.4)	0.581
>4	101 (40.6)	76 (39.4)	25 (44.6)	
L (median (IQR))	1.92 (1.60-2.45)	2.13 (1.75-2.61)	1.53 (1.36-1.83)	**<0.001**
N (median (IQR))	3.78 (3.00-4.73)	3.42 (2.86-4.22)	5.03 (4.42-6.22)	**<0.001**
M (median (IQR))	0.41 (0.34-0.52)	0.38 (0.31-0.47)	0.50 (0.41-0.58)	**<0.001**
HB (median (IQR))	135.0(128.3-141.0)	135.0(129.2-140.0)	133.5(126.1-142.3)	0.532
P (median (IQR))	239.0 (210-283)	238.0(210-278)	256.5(211-299)	0.293
ALB (median (IQR))	45.00 (43.00-46.50)	45.00 (43.00-46.30)	45.00 (43.00-46.78)	0.895
GLOB (median (IQR))	30.00 (27.60-33.00)	30.00 (27.30-33.00)	30.00(28.0-33.12)	0.621
BMI (median (IQR))	24.00 (21.80-26.70)	24.0 (21.60-26.60)	23.9(22.08-27.55)	0.242
PNI (median (IQR))	55.10 (52.30-57.50)	55.50 (52.90-58.10)	52.55(50.6-55.12)	**<0.001**
AGR (median (IQR))	1.48 (1.35-1.64)	1.50 (1.38-1.64)	1.46(1.33-1.61)	0.444
NLR (median (IQR))	1.87 (1.45-2.47)	1.66 (1.37-2.03)	3.20(2.75-3.64)	**<0.001**
PLR (median (IQR))	120.00(98-150)	113 (95-136)	162(130-206)	**<0.001**
MLR (median (IQR))	0.20 (0.16-0.27)	0.19 (0.15-0.22)	0.32(0.27-0.37)	**<0.001**
SII (median (IQR))	441 (329-630)	397 (299-509)	813(682-1001)	**<0.001**
SIRI (median (IQR))	0.77 (0.51-1.14)	0.64 (0.48-0.86)	1.52(1.36-2.08)	**<0.001**

Abbreviations: INPS, inflammation and nutrition prognostic score; IQR, interquartile range; ER, estrogen receptor; PR, progesterone receptor; HER-2, human epidermal growth factor receptor 2; HER-2 OE, HER-2 overexpression; L, lymphocyte; N, neutrophil; M, monocyte; Hb, hemoglobin; P, platelet; ALB, albumin; GLOB, globulin; BMI, body mass index; PNI, prognostic nutritional index; AGR, albumin-globulin ratio; NLR, neutrophil-lymphocyte ratio; PLR, platelet-lymphocyte ratio; MLR, monocyte-lymphocyte ratio; SII, systemic immune inflammation index; SIRI, systemic inflammation response index.

**Table 3:** .The relationship between different variables and OS for breast cancer patients with nonpCR.

Variables	Univariate analysis		Multivariate analysis	
	HR (95% CI)	*P* value	HR (95% CI)	*P* value
Age (≤49 vs. >49)	1.22 (0.77-1.94)	0.401		
Position (left vs. right)	1.31 (0.82-2.08)	0.253		
Menopause (no vs. yes)	1.36 (0.86-2.16)	0.194		
Parturition (0 vs. 1)	0.40 (0.22-0.74)	**0.004**	0.41 (0.22-0.77)	**0.006**
Parturition (0 vs. ≥2)	0.73 (0.38-1.43)	**0.362**	0.67 (0.33-1.34)	0.258
OS.INPS (≤0.3917 vs. >0.3917)	2.81 (1.75-4.51)	**<0.001**	2.41 (1.45-4.01)	**<0.001**
OS.L (≤1.48 vs. >1.48)	0.60 (0.35-1.02)	0.057		
OS.N (≤5.9 vs. >5.9)	2.38 (1.31-4.35)	**0.005**	1.67 (0.85-3.25)	0.134
OS.M (≤0.36 vs. >0.36)	1.31 (0.79-2.16)	0.292		
OS.HB (≤132 vs. >132)	0.63 (0.4-1.01)	0.053		
OS.P (≤313 vs. >313)	1.63 (0.9-2.98)	0.11		
OS.ALB (≤46.3 vs. >46.3)	1.28 (0.77-2.12)	0.348		
OS.GLOB (≤34.8 vs. >34.8)	1.67 (0.95-2.95)	0.077		
Clinical T stage (1 vs. 2)	0.59 (0.28-1.26)	**0.173**	0.68 (0.32-1.45)	0.315
Clinical T stage (1 vs. 3)	0.88 (0.38-2.05)	**0.771**	0.95 (0.41-2.21)	0.905
Clinical T stage (1 vs. 4)	5.Nn(1.37-19.5)	**0.015**	5.70 (1.43-22.8)	**0.014**
Clinical N stage (0 vs. 1)	1.48 (0.32-6.86)	0.615		
Clinical N stage (0 vs. 2)	1.19 (0.29-4.91)	0.81		
Clinical N stage (0 vs. 3)	1.5° (0.35-6.42)	0.588		
Clinical TNM stage (II vs. III)	0.92 (0.47-1.79)	0.806		
Molecular subtype (luminal A vs. B)	1.43 (0.71-2.87)	0.311		
Molecular subtype (luminal A vs. HER2-OE)	0.82 (0.34-1.96)	0.653		
Molecular subtype (luminal A vs. TNBC)	1.35 (0.59-3.09)	0.472		
ER (negative vs. positive)	1.28 (0.79-2.1)	0.317		
PR (negative vs. positive)	1.09 (0.68-1.73)	0.721		
HER-2 (negative vs. low expression)	1.07 (0.62-1.82)	0.818		
HER-2 (negative vs. positive)	0.78 (0.44-1.4)	0.408		
Ki-67 (<20% vs. ≥20%)	1.49 (0.92-2.4)	0.107		
P53 (negative vs. positive)	1.4 (0.85-2.29)	0.187		
Cycle (≤4 vs. >4)	1.23 (0.78-1.97)	0.374		

**Table 4:** The relationship between different variables and DFS for breast cancer patients with nonpCR.

Variables	Univariate analysis		Multivariate analysis	
	HR (95% CI)	*P* value	HR (95% CI)	*P* value
Age (≤49 vs. >49)	1.05 (0.73-1.5)	0.813		
Position (left vs. right)	1.08 (0.75-1.56)	0.679		
Menopause (no vs. yes)	1.24 (0.86-1.78)	0.255		
Parturition (0 vs. 1)	0.46 (0.28-0.76)	**0.002**	0.45 (0.27-0.76)	**0.003**
Parturition (0 vs. 2)	0.77 (0.44-1.34)	**0.352**	0.73 (0.41-1.28)	0.2683
DFS.INPS (≤0.4896 vs. >0.4896)	2.22 (1.5-3.27)	**<0.001**	1.84 (1.20-2.83)	**0.005**
DFS.L (≤1.39 vs. >1.39)	0.63 (0.39-1.03)	0.067		
DFS.N (≤5.47 vs. >5.47)	1.87 (1.2-2.91)	**0.006**	1.47 (0.90-2.41)	0.122
DFS.M (≤0.36 vs. >0.36)	1.59 (1.06-2.39)	**0.026**	1.18 (0.76-1.83)	0.459
DFS.HB (≤132 vs. >132)	0.74 (0.52-1.07)	0.110		
DFS.P (≤304 vs. >304)	1.38 (0.86-2.21)	0.185		
DFS.ALB (≤43 vs. >43)	1.43 (0.95-2.15)	0.091		
DFS.GLOB (≤28.5 vs. >28.5)	1.40 (0.94-2.1)	0.100		
Clinical T stage (1 vs. 2)	0.75 (0.4-1.42)	0.377		
Clinical T stage (1 vs. 3)	0.95 (0.47-1.93)	0.886		
Clinical T stage (1 vs. 4)	2.80 (0.78-10.05)	0.115		
Clinical N stage (0 vs. 1)	2.37 (0.54-10.51)	0.256		
Clinical N stage (0 vs. 2)	2.18 (0.54-8.92)	0.276		
Clinical N stage (0 vs. 3)	3.41 (0.82-14.2)	0.092		
Clinical TNM stage (II vs. III)	1.27 (0.71-2.27)	0.413		
Molecular subtype (luminal A vs. B)	1.72 (0.96-3.09)	0.066		
Molecular subtype (luminal A vs. HER-2 OE)	1.67 (0.86-3.23)	0.127		
Molecular subtype (luminal A vs. TNBC)	1.37 (0.68-2.75)	0.378		
ER (negative vs. positive)	1.00 (0.69-1.46)	0.980		
PR (negative vs. positive)	0.91 (0.63-1.3)	0.592		
HER-2 (negative vs. low expression)	1.18 (0.75-1.83)	0.476		
HER-2 (negative vs. positive)	1.34 (0.87-2.08)	0.189		
Ki-67 (<20% vs. ≥20%)	1.31 (0.9-1.91)	0.152		
P53 (negative vs. positive)	0.96 (0.64-1.46)	0.860		
Cycle (≤4 vs. >4)	1.18 (0.82-1.71)	0.371		

**Table 5:** AUC of inflammatory and nutritional variables for OS and DFS.

Variables	AUC (95% CI)	S.E.	*Z* statistic	*P* value
OS.INPS	0.615 (0.552-0.676)	0.032	3.601	**0.0003**
OS.BMI^∗^	0.511 (0.447-0.575)	0.035	0.318	0.7503
OS.PNI	0.532 (0.467-0.595)	0.018	1.728	0.0840
OS.AGR	0.535 (0.471-0.599)	0.025	1.427	0.1536
OS.NLR	0.610 (0.546-0.671)	0.031	3.503	**0.0005**
OS.PLR	0.546 (0.482-0.609)	0.031	1.487	0.1370
OS.MLR	0.539 (0.475-0.603)	0.026	1.546	0.1222
OS.SII	0.579 (0.515-0.641)	0.031	2.517	**0.0118**
OS.SIRI	0.563 (0.499-0.625)	0.029	2.202	**0.0277**
DFS.INPS	0.596 (0.532-0.658)	0.027	3.632	**0.0003**
DFS.BMI^∗∗^	0.534 (0.470-0.597)	0.032	1.074	0.2830
DFS.PNI	0.529 (0.465-0.593)	0.019	1.571	0.1161
DFS.AGR	0.536 (0.472-0.599)	0.021	1.750	0.0801
DFS.NLR	0.586 (0.522-0.648)	0.028	3.114	**0.0018**
DFS.PLR	0.537 (0.473-0.600)	0.032	1.158	0.2467
DFS.MLR	0.561 (0.497-0.624)	0.021	2.864	**0.0042**
DFS.SII	0.568 (0.504-0.630)	0.027	2.551	**0.0107**
DFS.SIRI	0.568 (0.504-0.631)	0.027	2.541	**0.0111**

^∗^The state variable for OS. BMI was OS; ^∗∗^ The state variable for DFS. BMI was DFS.

**Table 6:** Comparison of the prognostic ability of inflammatory and nutritional variables.

Variables	Difference of AUC	S.E.	95% CI	*Z* statistic	*P* value
OS.INPS vs. OS.BMI	0.1040	0.0497	0.007-0.202	2.094	**0.0363**
OS.INPS vs. OS.PNI	0.0838	0.0340	0.017-0.150	2.467	**0.0136**
OS.INPS vs. OS.AGR	0.0800	0.0416	-0.002-0.162	1.924	0.0544
OS.BMI vs. OS.PNI	0.0204	0.0401	-0.058-0.099	0.503	0.6113
OS.BMI vs. OS.AGR	0.0231	0.0401	-0.055-0.103	0.602	0.5473
OS.PNI vs. OS.AGR	0.0038	0.0308	-0.057-0.064	0.122	0.9025
OS.INPS vs. OS.NLR	0.0054	0.0109	-0.016-0.027	0.497	0.6193
OS.INPS vs. OS.PLR	0.0696	0.0377	-0.004-0.144	1.843	0.0653
OS.INPS vs. OS.MLR	0.0759	0.0292	0.019-0.133	2.603	**0.0092**
OS.INPS vs. OS.SII	0.0363	0.0216	-0.006-0.079	1.675	0.0939
OS.INPS vs. OS.SIRI	0.0525	0.0273	-0.001-0.106	1.925	0.0542
OS.NLR vs. OS.PLR	0.0641	0.0380	-0.010-0.139	1.688	0.0914
OS.NLR vs. OS.MLR	0.0705	0.0280	0.016-0.125	2.516	**0.0119**
OS.NLR vs. OS.SII	0.0308	0.0244	-0.017-0.079	1.262	0.2069
OS.NLR vs. OS.SIRI	0.0471	0.0258	-0.003-0.098	1.828	0.0676
OS.PLR vs. OS.MLR	0.0064	0.0363	-0.065-0.078	0.175	0.8610
OS.PLR vs. OS.SII	0.0333	0.0349	-0.035-0.102	0.954	0.3399
OS.PLR vs. OS.SIRI	0.0171	0.0395	-0.060-0.094	0.433	0.6654
OS.MLR vs. OS.SII	0.0397	0.0333	-0.026-0.105	1.193	0.2329
OS.MLR vs. OS.SIRI	0.0234	0.0209	-0.018-0.064	1.120	0.2625
OS.SII vs. OS.SIRI	0.0162	0.0314	-0.045-0.078	0.517	0.6054
DFS.INPS vs. DFS.BMI	0.0620	0.0421	-0.021-0.144	1.472	0.1411
DFS.INPS vs. DFS.PNI	0.0667	0.0304	0.007-0.126	2.193	**0.0283**
DFS.INPS vs. DFS.AGR	0.0599	0.0342	-0.007-0.127	1.753	0.0797
DFS.BMI vs. DFS.PNI	0.0047	0.0381	-0.070-0.079	0.124	0.9011
DFS.BMI vs. DFS.AGR	0.0021	0.0376	-0.072-0.076	0.055	0.9560
DFS.PNI vs. DFS.AGR	0.0068	0.0270	-0.046-0.060	0.252	0.8012
DFS.INPS vs. DFS.NLR	0.0102	0.0131	-0.016-0.036	0.777	0.4369
DFS.INPS vs. DFS.PLR	0.0593	0.0341	-0.007-0.126	1.741	0.0817
DFS.INPS vs. DFS.MLR	0.0351	0.0228	-0.010-0.080	1.542	0.1230
DFS.INPS vs. DFS.SII	0.0285	0.0199	-0.011-0.068	1.433	0.1518
DFS.INPS vs. DFS.SIRI	0.0280	0.0149	-0.001-0.057	1.875	0.0608
DFS.NLR vs. DFS.PLR	0.0491	0.0347	-0.019-0.117	1.418	0.1563
DFS.NLR vs. DFS.MLR	0.0250	0.0252	-0.024-0.074	0.992	0.3212
DFS.NLR vs. DFS.SII	0.0183	0.0224	-0.026-0.062	0.819	0.4129
DFS.NLR vs. DFS.SIRI	0.0178	0.0199	-0.021-0.057	0.894	0.3713
DFS.PLR vs. DFS.MLR	0.0242	0.0338	-0.042-0.090	0.716	0.4738
DFS.PLR vs. DFS.SII	0.0308	0.0295	-0.027-0.089	1.045	0.2960
DFS.PLR vs. DFS.SIRI	0.0313	0.0374	-0.042-0.105	0.838	0.4018
DFS.MLR vs. DFS.SII	0.0067	0.0262	-0.045-0.0579	0.253	0.8006
DFS.MLR vs. DFS.SIRI	0.0072	0.0221	-0.036-0.050	0.3250	0.7455
DFS.SII vs. DFS.SIRI	0.0006	0.0250	-0.048-0.049	0.0220	0.9824

## Data Availability

Due to the nature of this research, participants of this study did not agree for their data to be shared publicly, so supporting data is not available.

## Supplementary Materials

Supplementary Materials Table S1: the relationship between hematological parameters which were included into COX regression analysis, OS, and DFS. Certificate of English Editing: the first Certificate of English Editing. AJE editing certificate: the second Certificate of English Editing.
