# Supplementary material for: A Novel Inflammatory and Nutritional Prognostic Scoring System for Nonpathological Complete Response Breast Cancer Patients Undergoing Neoadjuvant Chemotherapy
Source: Dis Markers. 2022 Dec 16;2022:8044550. doi: 10.1155/2022/8044550 (PMC9788886; doi:10.1155/2022/8044550)
Supplement: Supplementary Materials — Table S1: the relationship between hematological parameters which were included into COX regression analysis, OS, and DFS. Certificate of English Editing: the first Certificate of English Editing. AJE editing certificate: the second Certificate of English Editing. [file 8044550.f1.zip › AJE editing certificate.pdf]

AJE®

Editing Certificate

This document certifies that the manuscript

A novel inflammatory and nutritional prognostic score system for non-pathologic complete response breast cancer patients undergoing neoadjuvant chemotherapy

prepared by the authors

Cong Jiang, Yuting Xiu, Shiyuan Zhang, Xiao Yu, Kun Qiao, Yuanxi Huang

was edited for proper English language, grammar, punctuation, spelling, and overall style by one or more of the highly qualified native English speaking editors at AJE.

This certificate was issued on **November 7, 2022** and may be verified on the AJE website using the verification code **1006-578B-9570-9B82-C0C8**.

Neither the research content nor the authors' intentions were altered in any way during the editing process. Documents receiving this certification should be English-ready for publication; however, the author has the ability to accept or reject our suggestions and changes. To verify the final AJE edited version, please visit our verification page at aje.com/certificate. If you have any questions or concerns about this edited document, please contact AJE at support@aje.com.

AJE provides a range of editing, translation, and manuscript services for researchers and publishers around the world. For more information about our company, services, and partner discounts, please visit aje.com.
