# Supplementary material for: A Novel Inflammatory and Nutritional Prognostic Scoring System for Nonpathological Complete Response Breast Cancer Patients Undergoing Neoadjuvant Chemotherapy
Source: Dis Markers. 2022 Dec 16;2022:8044550. doi: 10.1155/2022/8044550 (PMC9788886; doi:10.1155/2022/8044550)
Supplement: Supplementary Materials — Table S1: the relationship between hematological parameters which were included into COX regression analysis, OS, and DFS. Certificate of English Editing: the first Certificate of English Editing. AJE editing certificate: the second Certificate of English Editing. [file 8044550.f1.zip › Certificate of English Editing.pdf]

Certificate of

English Editing

This document certifies that the paper listed below has been edited to ensure that the language is clear and free of errors. The edit was performed by professional editors at *Eu-edit*. The intent of the author's message was not altered in any way during the editing process. The quality of the edit has been guaranteed, with the assumption that our suggested changes have been accepted and have not been further altered without the knowledge of our editors.

Title of the paper

[A novel inflammatory and nutritional prognostic score system for non-pathologic complete response breast cancer patients undergoing neoadjuvant chemotherapy]

Authors

[Cong Jiang, Yuting Xiu, Shiyuan Zhang, Xiao Yu, Kun Qiao, Yuanxi Huang]

Editor

Dr. Jonathan Busuttil from the university of Nottingham

About Eu-edit

Eu-edit offers professional English language editing and publication support services to authors engaged in over 500 areas of research. Through its community of experienced editors, which includes doctors, engineers, published scientists, and researchers with peer review experience, Eu-edit has successfully helped authors get published in internationally reputed journals. Authors who work with Eu-edit are guaranteed excellent language quality and timely delivery. **We also welcome cooperation from various journals. For further information, please contact the E-mail below**.

Contact us:

www.eu-edit.com Email: editor@euedit.com
