# Supplementary material for: A Novel Inflammatory and Nutritional Prognostic Scoring System for Nonpathological Complete Response Breast Cancer Patients Undergoing Neoadjuvant Chemotherapy
Source: Dis Markers. 2022 Dec 16;2022:8044550. doi: 10.1155/2022/8044550 (PMC9788886; doi:10.1155/2022/8044550)
Supplement: Supplementary Materials — Table S1: the relationship between hematological parameters which were included into COX regression analysis, OS, and DFS. Certificate of English Editing: the first Certificate of English Editing. AJE editing certificate: the second Certificate of English Editing. [file 8044550.f1.zip › Table S1.docx]

| **Table S1** The relationship between hematological parameters which were included into COX regression, OS and DFS. | | |
| --- | --- | --- |
| **Variables** | Better OS | Better DFS |
| Higher lymphocyte | yes | yes |
| Higher Hb | yes | yes |
| Higher neutrophil | no | no |
| Higher monocyte | no | no |
| Higher platelet | no | no |
| Higher Alb | no | no |
| Higher Glob | no | no |
| Higher INPS | no | no |
